# The pathogenesis of influenza in intact alveoli: virion endocytosis and its effects on the lung’s air-blood barrier

**DOI:** 10.3389/fimmu.2024.1328453

**Published:** 2024-01-26

**Authors:** Jaime L. Hook, Jahar Bhattacharya

**Affiliations:** ^1^ Lung Imaging Laboratory, Division of Pulmonary, Critical Care, and Sleep Medicine, Department of Medicine, Icahn School of Medicine at Mount Sinai, New York, NY, United States; ^2^ Global Health and Emerging Pathogens Institute, Department of Microbiology, Icahn School of Medicine at Mount Sinai, New York, NY, United States; ^3^ Department of Medicine, College of Physicians and Surgeons, Columbia University Medical Center, New York, NY, United States; ^4^ Department of Physiology and Cellular Biophysics, College of Physicians and Surgeons, Columbia University Medical Center, New York, NY, United States

**Keywords:** endocytosis, influenza A virus, blood-air barrier, pulmonary edema, acute lung injury

## Abstract

Lung infection by influenza A virus (IAV) is a major cause of global mortality from lung injury, a disease defined by widespread dysfunction of the lung’s air-blood barrier. Endocytosis of IAV virions by the alveolar epithelium – the cells that determine barrier function – is central to barrier loss mechanisms. Here, we address the current understanding of the mechanistic steps that lead to endocytosis in the alveolar epithelium, with an eye to how the unique structure of lung alveoli shapes endocytic mechanisms. We highlight where future studies of alveolar interactions with IAV virions may lead to new therapeutic approaches for IAV-induced lung injury.

## 1 Introduction

Lung infection by influenza A virus (IAV) is a major cause of global mortality. In the last century alone, four global IAV pandemics caused more than 50 million deaths (1, 2). In the modern era, seasonal IAV infections cause nearly half a million deaths every year (3, 4).

Death from IAV lung infection often results from lung injury (5, 6). Acute lung injury is defined by damage to lung alveoli that causes loss of alveolar barrier function, leading to airspace flooding with protein-rich edema fluid (7). Therapy for IAV-induced lung injury centers on antiviral drugs. But, antiviral drugs do not contain lung injury once it initiates (8–11) and are increasingly hindered by viral drug resistance (12, 13), creating a critical need for new approaches to therapy. The sizeable annual risk of a recurrent IAV pandemic (1, 14) makes that need not only critical, but urgent.

Endocytosis of IAV virions by alveolar cells is a central event on the path from lung infection to lung injury (15) and a target for antiviral drug development (16, 17). However, the understanding of endocytic mechanisms is largely informed by findings generated in systems that incorporate non-alveolar cells or that isolate alveolar cells from their normal microenvironment. The extent to which such systems represent IAV uptake mechanisms in alveoli *in vivo* may be limited, since unique aspects of alveolar structure that likely impact endocytic mechanisms are difficult to replicate *in vitro*.

Whereas previous reviews of IAV lung infection have focused on viral entry mechanisms derived from studies in cultured cells, here we will consider viral endocytosis in the alveolar epithelium from the standpoint of structural features of intact alveoli that likely impact endocytic mechanisms. Although alveolar macrophages also shape IAV lung pathogenesis, we will not address them in this review. By highlighting structural features of alveoli, we hope to stimulate new investigations of viral endocytosis in the alveolar epithelium that may lead to new therapeutic approaches for IAV-induced lung injury.

## 2 IAV virion inhalation into alveoli

Alveolar infection initiates when IAV virions are inhaled into the lung. IAV virions consist of a membrane envelope that surrounds the viral genome. The envelope contains host-derived lipids and proteins (18–20) and three viral proteins: hemagglutinin (HA), neuraminidase (NA), and the M2 ion channel (21). The viral matrix protein, M1 supports the envelope on its inner aspect. The genome consists of eight discrete, negative-sense, single-stranded RNA segments that each associate with viral nucleoprotein and an RNA-dependent RNA polymerase complex to form eight rod-like structures.

### 2.1 Determinants of virion inhalation into alveoli

**Figure 1:**
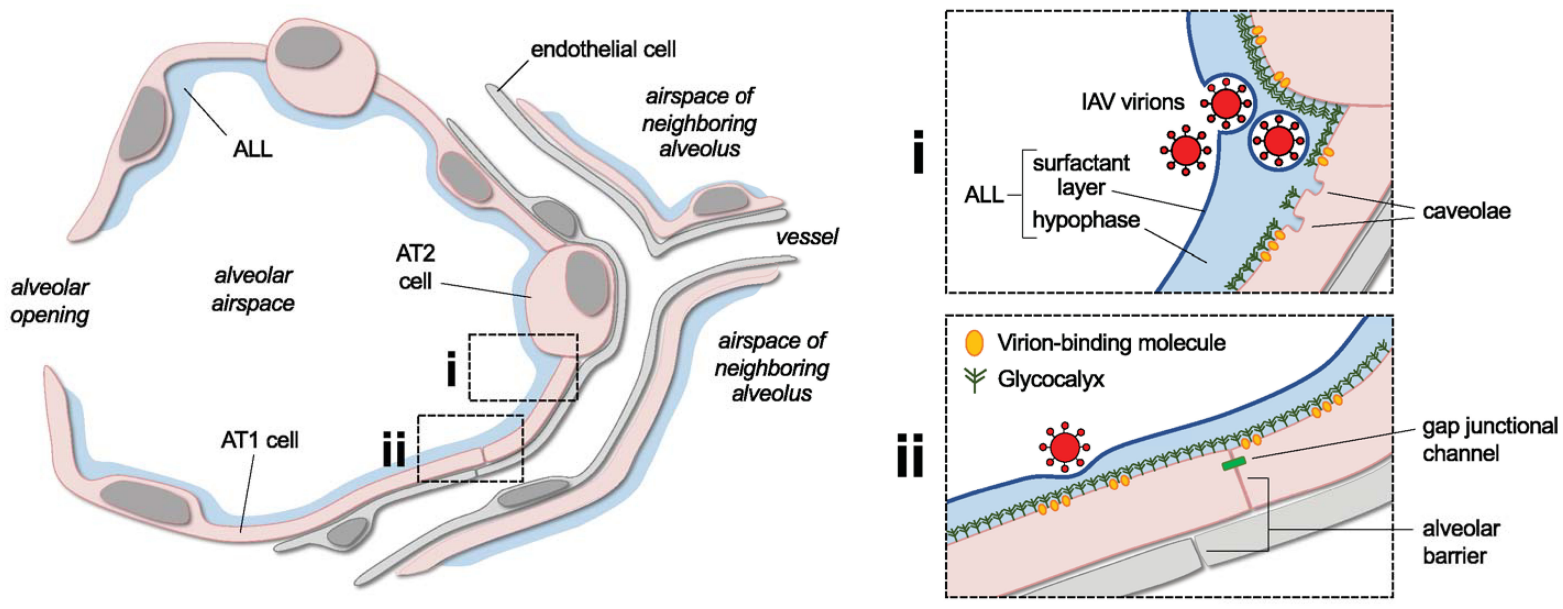
Overview of lung alveolar structure and selected features to be addressed in future research of the alveolar endocytosis of IAV virions. The cartoon shows a lung alveolus and parts of neighboring alveoli and microvessels. The alveolar lining layer (*ALL*) and alveolar epithelial type 1 (*AT1*) and 2 (*AT2*) cells are indicated. *Boxed cartoons at right* show enlarged views of *area i*, an alveolar corner, and *area ii*, a flat alveolar region. In *i*, the corner is depicted as a site of aggregation of inhaled IAV virions. Two virions are shown covered by a phospholipid film derived from the ALL’s surfactant layer. *Labels* highlight ALL components and plasma membrane *caveolae*, which are abundant in AT1 cells and located primarily near AT1-AT2 cell junctions. In *ii*, a virion interacts with the alveolar surface. *Symbols* approximate the epithelial glycocalyx and virion-binding molecules in alveolar epithelial membranes, and *labels* point out epithelial and endothelial components of the alveolar barrier and the intercellular gap junctional channels that conduct epithelial cell-cell signaling. See main text for discussion of how the depicted structural features might bear on the alveolar pathogenesis of IAV and may be addressed in future research. Note, in the cartoon, AT1 cells are shown as thicker than neighboring structures in order to emphasize the epithelium. However, publications including reference #153 indicate that the epithelium is, in fact, thinner than the interstitium and endothelial cells in alveoli of mammalian lungs. Size relationships between virions, AT1 cells, the glycocalyx, and the ALL are drawn approximately to scale.

Virions inhaled into the lungs are conducted through a series of branching airways of successively smaller diameter that transition distally from terminal bronchioles to acini, which include respiratory bronchioles, alveolar ducts, and alveoli. Alveoli are cup-like chambers that average 100-250 um in diameter in human lungs and comprise more than 95% – the vast majority – of the lung luminal surface (**Figure 1**) (22, 23). Despite their extensive surface area, alveoli are not the primary site of deposition for all inhaled particles. Only particles smaller than about 2 um diameter appear to be preferentially deposited in alveoli after inhalation (24). Studies of viral transmission suggest IAV virions are inhaled in liquid-containing particles as small as 1.5 um diameter (25–27) – a size that makes them likely to reach alveoli based on particle inhalation studies in human lungs (24). The possibility that inhaled IAV virions reach alveoli is supported by pathological studies of fixed lungs of rodents, primates, and humans with severe IAV infection, which identify virions, viral nucleic acids, and viral proteins associated with the alveolar epithelial type 1 (AT1) and type 2 (AT2) cells that line alveolar walls (28–31).

Whether inhaled particles, including IAV virions, reach alveoli is partly governed by the balance of convective and diffusive transport of gases in the lung. Broadly, convection refers to bulk transport of gases in the airways, while diffusion relates to gas mixing in acinar regions (32). The balance between convection and diffusion determines particle transport in non-lung model systems (33, 34). In healthy lungs, the convective-diffusive balance is neither static nor uniform. Rather, it varies with breathing pattern (24, 32) and across acinar geometries (35) that, themselves, change with age (36, 37) and vary by lung region (38, 39). At the acinar level, air flow represents a combination of convection and diffusion that generates chaotic gas mixing where inhaled and residual acinar gases meet (40–42). Going forward, better understanding is needed of how local and regional gas transport mechanisms affect the alveolar inhalation of IAV virions, since the inhalation of virion-sized particles into acini and alveoli is influenced by factors that affect the convective-diffusive balance (24, 35, 37, 43).

There is notably little understanding of how chronic lung diseases bear on the alveolar inhalation of IAV virions. Human aerosol inhalation studies (44–47) and computational models (48) suggest that airway obstruction due to asthma and chronic obstructive pulmonary disease (COPD) promotes the deposition of inhaled particles in central lung airways. By contrast, lung compliance changes related to aging and obesity may promote particle deposition in more distal lung regions, including alveoli (49–51). Clinical studies that associate severe IAV infection with aging and obesity – but not asthma or COPD (52–55) – raise the possibility that the tendency to inhale particles into distal lung regions promotes IAV pathogenesis. Experimental studies that relate airway obstruction, lung compliance, and IAV pathogenesis to the microanatomical location of inhaled particles might clarify these issues.

Finally, virion inhalation into alveoli is likely to be influenced by virion morphology. IAV virions assume spherical, bacilliform, or filamentous shapes in response to host and viral factors (56–58). Spherical and bacilliform virions range 120-150 nm in length, but filamentous virions can extend tens of microns long (56). Although studies of fixed lung tissue demonstrate the presence of all virion morphologies in IAV-infected human lungs (59, 60), the relevance of virion morphology to the alveolar pathogenesis of IAV is not clear. While electron microscopic data suggest that spherical and bacilliform virions associate with the alveolar epithelium (60), the relatively large size of filamentous virions may inhibit their inhalation into alveoli. Future studies might clarify how virion morphology affects alveolar inhalation dynamics and the extent to which inhaled filamentous virions initiate alveolar IAV infection.

### 2.2 Significance of alveolar structure for IAV virion inhalation

Once inhaled IAV virions reach alveoli, alveolar structure may influence how virions establish contact with alveolar walls. Alveolar structure is defined by flat tissue surfaces that alternate with curved, corner-like regions to form a unique tissue architecture. At flat septal regions, a thin band of tissue and surface lining liquid separates alveolar airspaces from the microvascular lumens of the adjacent capillary meshwork (23, 61). Flat septal regions converge to form more than five corner-like structures per alveolus (62), where the radius of corner curvature varies with the degree of lung inflation (63).

Alveolar structure might determine viral entry mechanisms by shaping how virions are presented to alveolar cells. Our group has shown, by live imaging of intact lungs, that alveolar corners are sites of accumulation for bacteria, inert particles, and liquid (64, 65). The extent to which virions also accumulate at alveolar corners is not clear. If such accumulation occurs, it might facilitate contact between IAV virions and AT2 cells, which are located primarily at alveolar corners (66, 67) and serve functions critical to alveolar homeostasis and injury responses.

Virion localization to corners or other alveolar sites may be determined by air flow patterns that arise from the unique structure of alveoli. Thus, an encounter with an alveolar opening causes fluid flowing in an airway to change from a linear flow pattern to a curved flow pattern, oriented into the alveolus (37, 40, 41). Simulation and experimental data show that the vectors of curved flow form a gradient, with vectors of lesser curvature intersecting with alveolar walls and vectors of greater curvature forming vortices in airspaces (37, 40, 41). Such rotational flow patterns may promote the deposition of particles on alveolar walls (37, 68), but the extent to which they determine virion deposition in alveoli is not clear. Better understanding in this area seems particularly important in the lungs of young children, whose developing acinar structure may promote rotational air flows and particle deposition in alveoli (37, 68), and who experience disproportionately high mortality from IAV infection (69–73).

Breathing also causes changes in alveolar structure that might further influence the deposition of inhaled IAV virions in alveoli. Using live imaging of intact, perfused lungs, our group showed that lung inflation causes a heterogeneous distribution of alveolar distention, with the greatest increase of alveolar septal length occurring at flat segments lined by AT1 cells (67). Interestingly, alveolar expansion is lost with aging in a manner that correlates with age-related increases of perialveolar and subpleural collagen density (74), suggesting that age-related changes in lung tissue have a constraining effect on alveolar micromechanics. Further research is needed to understand how breathing-induced changes of alveolar structure affect particle and virion localization in alveoli and to characterize how aging bears on these issues.

## 3 IAV virion attachment to the alveolar surface

To initiate viral entry, virions must stabilize on the alveolar surface by attaching to epithelial cells. The alveolar epithelium consists of two cell populations: large, thin AT1 cells that cover the bulk of the alveolar surface, and small, cuboidal AT2 cells. IAV attaches to both AT1 and AT2 cells of fixed lung tissue (75, 76), indicating that both cell types are susceptible to virion attachment.

### 3.1 Significance of the alveolar glycocalyx for IAV virion attachment

Attachment of IAV virions to host cells is mediated by interactions between the viral HA protein and sialic acids on the host cell surface. The HA protein protrudes from the virion surface and binds sialic acid moieties at a site on the HA head domain (77–79). Sialic acids are 9-carbon sugars typically found at the distal end of glycolipid and glycoprotein components of the host cell glycocalyx, a layer of carbohydrates, proteins, and lipids that covers the surface of all mammalian cells. Strong evidence supports the presence of an airspace-facing glycocalyx on the alveolar epithelial surface (80) that provides potential attachment sites for IAV virions inhaled into alveoli. This, coupled with the high density of HA protein on the virion surface – more than 300 per spherical virion (81) – seems to create ample opportunity for HA-sialic acid interactions in alveoli.

The alveolar pathogenesis of IAV is thought to derive, in part, from the configuration of chemical bonds between sialic acids and the galactose residues that connect them to glycocalyx glycolipids and glycoproteins (82–84). Two such configurations are recognized for their potential relevance to lung infection: an α2,3- link that connects the quaternary C2 carbon of sialic acid to the C3 carbon of galactose, and an α2,6- link that connects the C2 carbon of sialic acid to the C6 carbon of galactose. Although studies using erythrocyte agglutination show that IAV virions bind sialic acids bearing either linkage configuration (85, 86), IAV has strain-specific differences in linkage binding. Thus, the erythrocyte-agglutinating capacity is greater when mammalian-origin IAV strains are exposed to α2,6-bearing erythrocytes, and when avian-origin IAV strains are exposed to α2,3-bearing erythrocytes (85, 86). These findings suggest that IAV virions originating from mammalian versus avian sources have unique capacities for host cell attachment, depending on the sialic acid linkage present at the host cell surface. Notably, landmark studies show that the α2,3- linkage is highly expressed on the alveolar epithelium, and that avian viruses that preferentially bind the α2,3- linkage attach to alveoli of fixed lung tissue and infect the alveolar epithelium of lung tissue blocks (87, 88). Hence, the presence of the α2,3-galactose linkage on the alveolar epithelium is suggested to underlie the extreme severity of lung infection by avian IAV strains (83, 84) that cause severe lung injury with high mortality (89–93).

However, recent findings suggest the role of sialic acid linkages in the alveolar pathogenesis of IAV is more complex. The alveolar epithelium expresses sialic acids of both the α2,3- (76, 87, 94) and α2,6- (76, 87) linkage configurations, and specific linkage configurations may not be required for IAV attachment and infection in the lung (95, 96). In fact, virion attachment to cultured cells is enhanced by a mix of sialic acid linkage configurations (97), suggesting that linkage diversity, rather than expression of a single configuration, might determine virion binding to the alveolar epithelium. Moreover, virion affinity for specific linkages may change over time, since HA-sialic acid interactions are affected by point mutations in the HA head domain (77–79), and mutations accumulate within strains and occur frequently in the course of infection (98–100). The intra-infection mutation rate may be considerable, since longitudinal sequencing data from IAV-infected human subjects show that multiple mutations causing amino acid changes in HA occurred in nearly all subjects in the first days of IAV infection (100). The potential impact of such mutations is evident from reports showing that changes in only two amino acids alters the sialic acid binding specificity of HA (101, 102). Taking the sialic acid data together, we interpret that epithelial sialic linkage configurations likely impact the spatial profile of virion attachment on the alveolar surface. However, future research might address how α2,3- and α2,6- linkages distribute in alveoli and how linkage partitioning or blending affects virion attachment.

Emerging data suggest that non-sialic acid components of the host cell surface, such as carcinoembryonic cell adhesion molecule 6 (CEACAM6, also known as CD66c) and macrophage galactose-type lectin 1 (MGL1), contribute to virion attachment mechanisms (103, 104). CEACAM6 and MGL1 may determine virion attachment to alveoli *in vivo*, since they are each expressed by the alveolar epithelium (105, 106). High-throughput approaches have identified other novel potential attachment mediators in alveolar epithelial-like A549 cells (107), but their relevance to virion attachment in alveoli has yet to be defined.

The extent to which virion attachment leads to endocytosis in intact alveoli may be determined by glycocalyx shedding. Intranasal instillation of IAV in mice induces shedding of the lung epithelial glycocalyx (108). Shedding releases glycocalyx components into airspaces (108), leading to a reduction of glycocalyx height and density (109). Shedding might defend against virion endocytosis by coating virions in shed matrix components and displacing them from the epithelial surface, but direct evidence for this possibility is lacking. Human studies that show evidence of glycocalyx shedding in severe lung infection (110) support the potential relevance of glycocalyx shedding to the alveolar pathogenesis of IAV. Further research might clarify how shedding of the alveolar epithelial glycocalyx affects viral entry in alveoli.

### 3.2 Significance of the alveolar lining layer for IAV virion attachment

The alveolar wall is covered by a lining layer that may provide a physical barrier to virion-glycocalyx interactions. The alveolar lining layer consists of an aqueous hypophase underlying a film of surfactant phospholipids and proteins at the air-liquid interface. Although the lining layer covers the alveolar wall in a continuous fashion, its depth varies across the alveolar surface (61). Thus, thin portions of the lining layer project an average height of 140 nm from the alveolar wall on flat surfaces of rat alveoli, and thicker portions project 1,000 nm or more at alveolar corners (61). The lining layer completely submerges alveolar epithelial cells and alveolar macrophages even at its thinnest parts (61), but whether the lining layer entirely submerges the glycocalyx remains unclear. Data generated by thorium dioxide-based electron microscopy show that the epithelial glycocalyx height also varies across alveolar regions, with its shortest level on the surfaces of AT1 cells that line flat alveolar walls (109). The extent to which the alveolar lining layer shields the epithelial glycocalyx from virion attachment in alveoli *in vivo* remains uncertain, but it may have implications for viral entry mechanisms, particularly on flat alveolar surfaces.

Surfactant proteins and phospholipids might also inhibit virion attachment to the alveolar epithelium. Reports indicate that particles inhaled into alveoli of rodent lungs become submerged in the alveolar lining layer and coated by a phospholipid film (111). There is little understanding of how surfactant phospholipids interact with inhaled IAV virions in alveoli. Since surfactant proteins enhance the uptake of IAV virions by macrophages *in vitro* (112), surfactant-virion interactions in alveoli *in vivo* might promote virion phagocytosis by alveolar macrophages. A protective role for surfactant proteins is supported by mouse models of IAV infection, in which mice with deficiencies of surfactant proteins A and D have impaired viral clearance from the lung (113, 114). Recombinant surfactant protein D binds the viral HA protein to protect against IAV uptake and replication in lungs of IAV-infected mice (115, 116), supporting the therapeutic potential of surfactant proteins in IAV infection.

The aqueous portion of the alveolar lining layer might further limit virion attachment to alveolar walls. Our group has shown that, under baseline conditions, the aqueous hypophase of the alveolar lining layer is secreted continuously by the alveolar epithelium (65, 117). The secretion generates a flow of alveolar wall liquid on the alveolar surface that clears particles from alveoli by convective transport (117). Alveolar wall liquid secretion also promotes the alveolar clearance of *S. aureus* bacteria (64, 65), but whether the secretion clears IAV virions from alveoli, thereby blocking their endocytosis, is not clear. Since we reported recently that IAV disrupts alveolar wall liquid secretion (65), the capacity of the secretion to clear virions may change in IAV-infected lungs over the course of lung infection.

## 4 IAV virion endocytosis by the alveolar epithelium

Binding of IAV virions to sialic acids is neither necessary (118) nor sufficient (119) to induce endocytosis by cultured cells, suggesting that the endocytosis is mediated by non-sialic acid interactions between virions and host membrane proteins. Here, we will address the known pathways of IAV virion endocytosis that are supported by evidence generated in alveoli, cultured alveolar cells, or cultured alveolar-like cells. Broadly, reports indicate that IAV virions exploit both clathrin-dependent and clathrin-independent endocytic pathways.

### 4.1 Clathrin-dependent mechanisms in alveoli

Evidence supports clathrin-mediated endocytosis (CME) as a mechanism of IAV virion uptake by cultured cells of non-alveolar origin (120–123). Briefly, CME is triggered by interactions between extracellular cargo and the plasma membrane phosphoinositide, PI(4,5)P2, that lead to binding of PI(4,5)P2 by the cytosolic adaptor protein 2 (AP2) (124, 125). AP2 stimulates the assembly of a protein complex that includes the scaffolding protein, clathrin. Lateral enlargement of the clathrin complex along the inner plasma membrane surface forms a polyhedral clathrin-containing “coat” that, along with polymerization of an actin filament network, induces plasma membrane curvature to form an endocytic vesicle. The developing vesicle is progressively constricted at the vesicle neck, then released into the cytosol by the cooperative activity of dynamin, an intracellular GTPase, and proteins of the Bin/amphiphysin/Rvs (BAR) domain family.

IAV virions are taken up by CME in cultured alveolar epithelial-like cells. Thus, data generated in cultured, alveolar epithelial-like A549 cells indicate that IAV virions interact with the epidermal growth factor receptor (EGFR), free fatty acid receptor 2 (FFAR2), or transferrin receptor 1 (TfR1) to initiate endocytosis by CME (126–128). Importantly, genetic inhibition of each protein blocks IAV entry and replication (126–128). Although these findings provide strong evidence that EGFR, FFAR2, TfR1, and CME drive viral entry mechanisms in A549 cells, future studies are needed to determine their roles the alveolar epithelium, since A549 cells are a cancer cell line (129) that exhibit features not shared with either AT1 or AT2 cells (129, 130). However, single cell transcriptomic datasets indicate EGFR, FFAR2, and TfR1 are each expressed by the alveolar epithelium of human lungs (131), bolstering the potential relevance of these proteins as initiators of CME for IAV virion endocytosis in alveoli.

### 4.2 Clathrin-independent mechanisms in alveoli

Clathrin-independent endocytic pathways may also mediate the alveolar epithelial uptake of IAV virions. In non-alveolar epithelial cells, IAV virions are taken up by multiple clathrin-independent pathways including caveolae-mediated endocytosis (132), macropinocytosis (133, 134) and a mechanism that involves the small GTPase, Cdc42 (135). Few studies have addressed whether such clathrin-independent pathways facilitate the endocytosis of IAV virions in alveoli. There is evidence that, in cultured alveolar epithelial-like A549 cells, the virion endocytic pathway initiated by EGFR that was discussed earlier in this review proceeds not only through CME, but also through an endocytic pathway that involves the caveolin-1 protein (126). While the mechanistic details of the caveolin-1 pathway remain uncertain, support for caveolin-1 as a potential mediator of virion endocytosis in alveoli comes from studies of uptake mechanisms of non-virion cargo. Genetic inhibition of caveolin-1 blocks endocytosis of *P. aeruginosa* (136) and nanoparticles (137, 138) in cultured alveolar epithelial-like cells, indicating caveolin-1 is critical to the endocytic mechanisms. Immunolabeling studies that identify caveolin-1 in AT1 and AT2 cells of fixed rat lungs (139) lend plausibility to a role for caveolin-1 in alveolar function. Taken together, these data support the possibility that caveolin-1 mediates endocytosis of IAV virions in the alveolar epithelium, but further studies are needed to draw this conclusion definitively.

Caveolin-1 is known to associate with plasma membrane domains called caveolae, but endocytic pathways mediated by caveolin-1 protein may occur independently of membrane caveolae in alveoli. Caveolae are plasma membrane regions defined by their pit-shaped architecture and specific lipid and protein composition, which includes core structural proteins such as caveolin-1. Membrane caveolae are traditionally thought to mediate endocytosis (140–143), but emerging views call into question the extent to which caveolae participate in endocytic mechanisms (144–146). This issue is relevant to alveoli because expression patterns of caveolin-1 protein and membrane caveolae are discordant in cells of the alveolar epithelium. Thus, caveolin-1 protein is expressed by both AT1 and AT2 cells (139, 147–151), but membrane caveolae are identified only in AT1 cells (139). Yet, immunofluorescence studies of cultured AT2 cells show caveolin-1 protein colocalizes with albumin taken up by endocytosis (152), suggesting caveolin-1 participates in endocytic mechanisms in alveolar epithelial cells that lack membrane caveolae.

In AT1 cells, the extent to which membrane caveolae and caveolin-1 protein mediate endocytosis remains unclear. Better understanding of their roles may be important, since alveolar epithelial expression of caveolin-1 protein occurs primarily in AT1 cells (139, 147–151), and AT1 cell membranes contain numerous caveolae. In fact, Gil et al. determined AT1 cells contain more than 150 vesicular structures per um^2^ of the luminal plasma membrane surface in unchallenged rabbit lungs (153). Even though immunolocalization studies suggest only some of the vesicular structures are caveolae (139), the calculations of Gil et al., taken in the context of the extensive surface area of AT1 cells (154), suggest caveolae number more than half a million in the AT1 cells that line alveolar walls. Immunolabeling studies identify caveolin-1 protein near AT1-AT2 cell junctions (139), and electron microscopic images demonstrate few caveolae in AT1 cell regions that line flat alveolar septa (23, 153, 155). This paucity of caveolin-1 protein and membrane caveolae at flat alveolar septa suggests that the endocytosis of IAV virions occurs at non-septal regions of the intact alveolus.

### 4.3 Virion motility across the alveolar surface

Reports indicate virion-epithelial interactions that lead to endocytosis are spatially and temporally dynamic and might localize virions to membrane sites conducive to endocytosis. After virion HA binds to host sialic acids, cooperative activity of HA and the viral neuraminidase (NA), an enzyme that cleaves sialic acids to release them from the glycocalyx, generates a series of sialic acid binding and cleavage events that induces IAV virion motility across a sialic acid-coated surface (156–158). If such motility occurs on the alveolar surface, it might move virions into proximity to EGFR, FFAR2, or TfR1. IAV virions also induce movement of host endocytic factors to sites of virion attachment. In cultured kidney epithelial-like cells, IAV virions induce clathrin accumulation at virion binding sites (120), perhaps promoting CME. Virion-induced plasma membrane clustering of the lipid raft ganglioside, GM1 (126) may lead to interactions between IAV virions and GM1 that are pro-endocytic (159). In alveoli, we do not identify evidence to support virion motility or to indicate whether such motility, if it occurs, promotes endocytosis.

## 5 Post-endocytic events that lead to alveolar barrier dysfunction

After endocytosis, IAV virions undergo a number of processing events that establish host cell infection: (i) endosomal trafficking; (ii) fusion with endosomal membranes to release viral nucleoproteins into the host cell cytosol; (iii) nucleoprotein trafficking to the host cell nucleus; (iv) viral RNA replication and transcription; (v) viral mRNA export to the cytosol; (vi) mRNA translation and viral protein processing; and (vii) virion formation and release via assembly and budding of viral proteins and nucleoproteins at the host cell plasma membrane. Here, we will briefly address the consequences of IAV virion endocytosis for alveolar function, specifically function of the alveolar barrier.

### 5.1 Alveolar barrier loss mechanisms

IAV lung infection causes dysfunction of the alveolar barrier, leading to pulmonary edema formation (160–166). Under baseline conditions, the alveolar barrier restricts the passage of proteins and small molecules from microvessels into airspaces to maintain airspace patency. The barrier is comprised of alveolar epithelial and microvascular endothelial cells that share a basement membrane (167). Barrier permeability is regulated at sites of contact between adjacent epithelial or adjacent endothelial cells by tight junctional protein complexes that incorporate claudins, occludins, junctional adhesion molecules, and cytoskeleton-binding adaptor proteins (168). Epithelial cells are the chief regulators of alveolar barrier permeability (169–171) due to the high density of junctional complexes at epithelial intercellular surfaces (172) and the large size of AT1 cells (154), which minimizes the total junctional surface area (173).

Mechanisms of alveolar barrier loss may include direct effects of IAV infection on alveolar epithelial barrier proteins. In cultured A549 cells, H1N1 IAV exposure causes loss of the tight junction proteins, occludin and ZO-1, and the adherens junction protein, E-cadherin by inhibiting junctional protein transcription (174). Although junctional protein loss is also reported in A549 cells exposed to H5N1 IAV, it results from protein degradation via the E3 ubiquitin ligase, Itch (93). Inhibition of the upstream kinase, TAK1 restores junctional protein expression in H5N1-exposed cultured cells, and intraperitoneal pretreatment with a TAK1 inhibitor restores alveolar junctional protein expression and increases survival in H5N1-infected mice (93). Findings by others confirm loss of the tight junction protein, claudin-4, in cells of the distal lung epithelial cancer line, NCI-H441, after exposure to either H5N1 or H1N1 IAV (175). Taken together, these findings provide evidence that IAV causes loss of alveolar epithelial junctional proteins by pre- and post-translational mechanisms, leading directly to loss of alveolar barrier function.

Indirect mechanisms may also mediate IAV-induced alveolar barrier loss. Reports indicate IAV induces proinflammatory signaling in alveolar epithelial and alveolar epithelial-like cells (176–184), leading to inflammation-induced alveolar damage (185–188). Numerous studies support the notion that IAV induces cell death in the alveolar epithelium (31, 161, 164, 189–191) and in cultured alveolar epithelial and alveolar epithelial-like cells (166, 192, 193), contributing to barrier loss. Once edema forms, IAV-induced alterations of alveolar epithelial ion channel function may further dysregulate alveolar liquid dynamics to potentiate the edema response (160, 194–198). Together, these data support the capacity of IAV to induce alveolar barrier loss by indirect effects on alveolar barrier function.

Although we do not address them in this review, other mechanisms may contribute to barrier loss and edema formation in IAV-infected lungs but are not well understood. For example, the interplay between surfactant, surface tension, and barrier permeability in alveoli of IAV-infected lungs may have implications for edema formation and virion distribution. Thus, IAV infection of AT2 cells (199, 200) may cause loss of surfactant secretion, leading to increase of alveolar surface tension and edema formation by a mechanism proposed by Pattle (201) and Clements (202) and supported by experimental data (203, 204). How this edema formation might affect the subsequent distribution of virions in alveoli is not clear. In addition, the specific mechanisms by which different IAV strains affect alveolar barrier function remain unclear, but they may relate to viral factors directly (205, 206) or to how viruses interact with the host (207–211). New insights in this area might address the disproportionately high morbidity and mortality caused by pandemic and avian IAV strains (212, 213). Finally, a growing literature supports a role for circadian rhythms in lung inflammation and repair in lungs that are mechanically ventilated (214) and IAV-infected (215–217). Better understanding of how sleep and circadian rhythm disruption affects alveolar responses to IAV may lead to new therapeutic approaches for infection-induced critical illness (218, 219).

### 5.2 Significance of alveolar communication pathways for barrier loss

Recent advances highlight the potential for cell-cell communication pathways present in intact alveoli to amplify the barrier dysfunction that follows virion endocytosis (220). Necropsy studies of human lungs with severe IAV infection identify little, if any, viral nucleic acids, protein, or cytopathic changes in the alveolar epithelium at sites of alveolar damage and barrier loss, at the same time that such evidence of active IAV infection is detectable elsewhere in the lungs (191, 221–224). Although these findings might reflect the tendency of tissue damage to linger after the resolution of viral infection, alternatively, they could reflect tissue damage that occurred due to spread of alveolar damage signals from infected to uninfected alveoli.

Interferon signaling might spread alveolar damage and barrier dysfunction in intact alveoli of IAV-infected lungs. Interferon signals spread from IAV-infected to uninfected cells in culture (225) and in alveoli (226), and interferon exposure causes loss of tight junctional proteins and barrier permeability in cultured intestinal epithelial-like cells (227, 228). These data raise the possibility that barrier-deteriorating interferon signals spread from infected to uninfected alveoli in IAV-exposed lungs, such that alveolar epithelial cells need not take up IAV virions directly to lose barrier function. A critical role for interferon signaling in influenza infection is supported by human data that identify genetic mutations in the interferon system in patients with influenza-induced critical illness (229–231).

Gap junctional communication in intact alveoli might also expand the scope of IAV-induced barrier loss. We have shown connexin 43-containing gap junctional channels conduct cytosolic Ca^2+^ signals in the alveolar epithelium (64, 232, 233) that, in *S. aureus-*infected alveoli, spread barrier loss from infected to uninfected alveolar regions (64). Since reports indicate IAV infection induces cytosolic Ca^2+^ increases in alveolar epithelial-like A549 cells (234, 235), IAV might also induce spread of cytosolic Ca^2+^ signals that amplify the barrier loss effects of virion endocytosis. Evidence is needed to determine whether this occurs in intact alveoli.

## 6 Conclusion

This review highlights key mechanistic steps underlying virion endocytosis in alveoli of IAV-infected lungs. Evidence from fixed lung tissue supports the notion that inhaled IAV virions can attach to AT1 and AT2 cells of the alveolar epithelium. The attachment mechanisms may involve virion interactions with sialic acids of the alveolar glycocalyx and alveolar epithelial surface proteins, such as CEACAM6 and MGL1. Data generated in cultured cells indicate that, after attachment, IAV virions are taken up by clathrin-dependent and clathrin-independent endocytic mechanisms. In this regard, EGFR-, FFAR2-, TfR1, and caveolin-1 may drive alveolar endocytic responses and provide new opportunities for the development of host-directed therapies that block viral entry at its earliest mechanistic steps. Finally, endocytosis of IAV virions leads to alveolar barrier permeability, and evidence derived from cultured cells suggests that the permeability responses might result from loss of alveolar barrier proteins. Targeting these responses may likewise yield new therapeutic approaches.

However, we point out that the unique structure of intact alveoli raises numerous, as yet unstudied questions about the mechanisms by which IAV virions are inhaled into alveoli, attach to the alveolar surface, and are taken up by the alveolar epithelium. For example, whether virions accumulate at alveolar corners is not clear but may determine which cells of the alveolar epithelium are exposed to and take up the virus. The extent to which virion attachment to the alveolar surface is opposed by glycocalyx shedding, surfactant binding, and alveolar wall liquid secretion in uncertain but may bear on the efficacy of virion attachment and internalization in intact alveoli. Alveolar cell-cell communication pathways may spread virus-induced interferon and Ca^2+^ responses, perhaps negating the need for virion endocytosis in barrier loss mechanisms. Thus, new insights are needed to generate a more fundamental understanding of IAV lung pathogenesis mechanisms that takes into account these unique structural features of intact alveoli. A better understanding of how alveolar structure shapes alveolar interactions with IAV may lead to novel approaches to therapy for IAV-induced lung injury that disrupt virion endocytosis and the barrier dysfunction that follows.

## Author contributions

JH: Conceptualization, Writing – original draft, Writing – review & editing. JB: Conceptualization, Writing – review & editing.
